# Primary hyperlipidemia in children: clinical, biochemistry characteristics and outcome

**DOI:** 10.1186/1687-9856-2015-S1-P124

**Published:** 2015-04-28

**Authors:** Nguyen Ngoc Khanh, Vu Chi Dung, Bui Phuong Thao, Can Thi Bich Ngoc, Nguyen Thi Hoan, Nguyen Phu Dat

**Affiliations:** 1 National Hospital of Pediatrics, Hanoi, Vietnam

## Background

Primary hyperlipidemia is genetic dyslipoproteinemia. Without any intervention, cardiovascular diseases and acute pancreatitis may be occurred. The detection and appropriate management of pediatric hyperlipidemia can have a significant impact upon the disease course and can prevent complications.

## Objects

to describe the clinical and biochemical characteristics of hyperlipidemia in Vietnamese children and to evaluate outcome of treatment.

## Patients and methods

From 2007 to 2013, 30 children with primary hyperlipidemia were recruited and were treated with diet and/or lipid-lowering drug therapy at the National Hospital of Pediatrics, Hanoi, Vietnam. Clinical symptoms and biochemical finding, outcome of treatment were studied. Results: Among 30 cases from 28 families, 8 patients were mixed hyperlipidemia (MHL), 13 patients were hypertriglyceridemia (HT) and 9 patients were hypercholesterolemia (HC). Mean age of diagnosis was 5.5 years (1 month – 16 years). The rate of male/female was 13/17. Clinical manifestations included hepatomegaly (4 cases), xanthemas in the knees and elbows (5 cases), “creamy” blood (21 cases). Twenty cases were clinical asymptomatic. 8/28 patients had family history with hyperlipidemia and cardiovascular diseases. Serum cholesterol levels of HC group was 9.2 ± 4 mmol/l. Serum triglyceride level of HT group was 23.6 ± 9.9 mmol/l. MHL group had hypercholesterolemia (12.1 ± 4.5 mmol/l) and hypertriglyceridemia (20.3 ± 10.5 mmol/l). After interventions, HT group had the best outcome with serum triglyceride level was 10.1 ± 4.6 mmol/l, next to MHL group with serum cholesterole level was 5.8 ± 1.8 mmol/l, and serum triglyceride level was 9.5 ± 5.2 mmol/l; finally, serum cholesterole level of HC group was 12.4 ± 5.5 mmol/l. Five infants with HT had the best outcome of treatment: serum triglyceride level decreased from 19 - 57.6 mmol/l to 5 - 10 mmol/l. Two patients with HC had the worsen results (unchanged blood lipid level).

## Conclusions

Primary hyperlipidemia had poor clinical manifestations and good results of treatment. Screening for primary hyperlipidemia help to prevent premature cardiovascular diseases.

